# The Impact of Torso Signal Processing on Noninvasive Electrocardiographic Imaging Reconstructions

**DOI:** 10.1109/TBME.2020.3003465

**Published:** 2021-01-20

**Authors:** Laura R. Bear, Yesim Serinagaoglu Dogrusoz, Wilson Good, Jana Svehlikova, Jaume Coll-Font, Eelco van Dam, Rob MacLeod

**Affiliations:** IHU-LIRYC, Fondation Bordeaux Université, Université de Bordeaux and INSERM, U1045 CRCTB, 33000 Bordeaux, France; Electrical and Electronics Engineering Department.; Department of Biomedical Engineering and SCI Institute, University of Utah.; Institute of Measurement Science, Slovak Academy of Sciences.; Computational Radiology Laboratory, Children’s Hospital.; Peacs BV, Nieuwerbrug aan den Rijn.; Department of Biomedical Engineering and SCI Institute, University of Utah.

**Keywords:** ECGI, inverse problem, PVC, Signal processing, Torso Tank

## Abstract

**Goal:**

To evaluate state-of-the-art signal processing methods for epicardial potential-based noninvasive electrocardiographic imaging reconstructions of single-site pacing data.

**Methods:**

Experimental data were obtained from two torso-tank setups in which Langendorff-perfused hearts (n = 4) were suspended and potentials recorded simultaneously from torso and epicardial surfaces. 49 different signal processing methods were applied to torso potentials, grouped as i) high-frequency noise removal (HFR) methods ii) baseline drift removal (BDR) methods and iii) combined HFR+BDR. The inverse problem was solved and reconstructed electrograms and activation maps compared to those directly recorded.

**Results:**

HFR showed no difference compared to not filtering in terms of absolute differences in reconstructed electrogram amplitudes nor median correlation in QRS waveforms (p > 0.05). However, correlation and mean absolute error of activation times and pacing site localization were improved with all methods except a notch filter. HFR applied post-reconstruction produced no differences compared to pre-reconstruction. BDR and BDR+HFR significantly improved absolute and relative difference, and correlation in electrograms (p < 0.05). While BDR+HFR combined improved activation time and pacing site detection, BDR alone produced significantly lower correlation and higher localization errors (p < 0.05).

**Conclusion:**

BDR improves reconstructed electrogram morphologies and amplitudes due to a reduction in lambda value selected for the inverse problem. The simplest method (resetting the isoelectric point) is sufficient to see these improvements. HFR does not impact electrogram accuracy, but does impact post-processing to extract features such as activation times. Removal of line noise is insufficient to see these changes. HFR should be applied post-reconstruction to ensure over-filtering does not occur.

## I. INTRODUCTION

**N**ONINVASIVE electrocardiographic imaging (ECGI) has been developed to provide high-resolution images of cardiac electrical activity. ECGI is increasingly being used to guide ablation therapy, such as in the identification of the origin of premature ventricular contractions (PVCs) or epicardial exit sites of ventricular arrhythmias [1]–[4].

Despite the increase in clinical adoption, previous validation studies of epicardial potential-based methods have shown varying results with respect to accuracy. Mean localization errors for known pacing sites in studies with *in-vivo* animal and human subjects have ranged from 6–50 mm [5]–[8]. Recent clinical validation studies have also shown large variability in activation map reconstruction accuracy (correlation from −0.68 to 0.82 in one study [9], 0.29 to 0.80 in another [10]). The variability in accuracy seen between different centers may be linked to the different inverse or post-processing methods used by each group. However, given that large variability exists even in single center studies using the same inverse method pipeline, it is unlikely the inverse or post-processing methods are the sole source of this variability.

ECGI is an ill-posed problem meaning any error in the model can have a drastic influence on the solution. This error can come from the presence of noise in the body surface potentials not related to the underlying cardiac electrical activity (i.e. power line interference, channel noise etc.), or the definition of the forward model itself. Signal processing can be used to minimize the impact of noise in the ECG including filtering, baseline drift removal and/or signal averaging. However, over-processing of signals may introduce further error by removing or distorting potentially important information relating to the cardiac electrical activity that would impact the ECGI reconstruction.

While guidelines exist for the processing of the standard 12-lead ECG signals to ensure appropriate interpretation [11], there is currently no consensus on the best signal processing methods for use with ECGI. Indeed, nearly every group implementing ECGI uses different signal processing methods. This is partially also due to the non-standardized acquisition environments, with differing hardware, electrode configurations and noise levels potentially requiring different processing methods.

The effect of noise on ECGI has previously been assessed by adding noise (typically Gaussian and high frequency) to forward simulated ECGs, then solving the inverse problem to determine their effects. These computational studies have been used to demonstrate that there is an increase in error in inverse reconstructions with increasing noise levels, as would be expected [12], [13]. Furthermore, the increase in inverse reconstruction error seen with signal noise is greater in more complex forward models incorporating inhomogeneous structures, meaning their accuracy becomes as good as a more simply defined homogeneous model [14], [15]. The effect of removing noise using different filtering methods on inverse reconstructions in an experimental setting has yet to be assessed.

The objective of this study was to collect the signal processing methods used by different ECGI centers and test their effects on a standard ECGI approach in the reconstruction of single-site pacing data, mimicking PVC’s seen in clinics. We have used data from two independent experimental setups to ensure the results are not biased to a single acquisition environment.

## II. EXPERIMENTAL DATA SETS

**Fig. 1.**
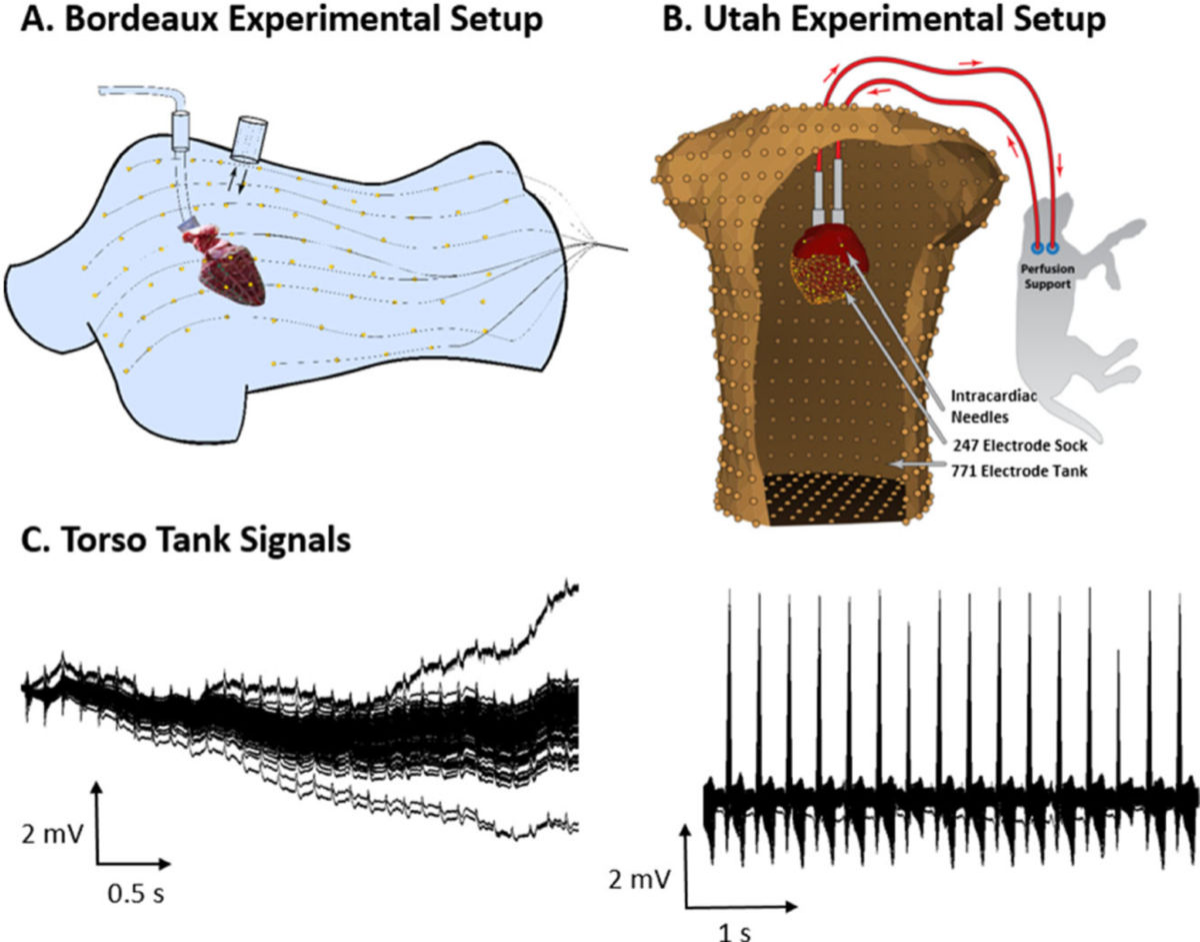
Torso Tank experimental setups used to obtain (A) two Bordeaux and (B) two Utah data sets. (C) The four sets were selected: one including “Noisy” torso signals being highly contaminated by high frequency noise and base line drift (left) and three with “Clean” torso signals with minimal high frequency noise or baseline wander (right).

In this study, experimental data came from two different torso tank experimental setups (Fig. 1A and B), from IHU-Liryc (Bordeaux, France) and the CRVTI/SCI Institutes (Salt Lake City, Utah), available at http://edgar.sci.utah.edu/. Four data sets were selected, three with comparable levels of noise that were relatively “clean” with little to no high frequency noise or baseline wander (example in Fig. 1C right), and one “Noisy” data set being highly contaminated by high frequency noise and base line drift (Fig. 1C left). This higher level of noise was likely due to environmental factors (i.e. a moving reference cable or additional noise from other nearby apparatus) and was included as this is a common problem seen in clinical recordings.

### A. Bordeaux Data

The experimental protocol used to obtain two of the four data sets has previously been described in [16] and is summarized below. All experimental procedures were approved by the Directive 2010/63/EU of the European Parliament on the protection of animals used for scientific purposes and the local ethical committee.

Excised pig hearts were perfused in Langendorff mode with 100% oxygenated Tyrode’s solution (pH 7.4, 37 °C). An epicardial electrode sock (108 electrodes with inter-electrode spacing 9.9 ± 2.2 mm) was attached to the ventricles and bipolar pacing leads to the RV apex. The hearts were transferred to a human-shaped torso tank with 128 electrodes (inter-electrode spacing 66 ± 24 mm) embedded in the surface (Fig. 1A). Tank and sock signals were referenced to an electrode at the bottom of the tank and recorded simultaneously (BioSemi, the Netherlands) at 2048 Hz for approximately 30 s during RV pacing. Afterwards, a 3D fluoroscopy (Artis, Siemens) was used to obtain the position of the epicardium and electrodes with respect to the tank.

### B. Utah Data

The experimental protocol used to obtain the other two data sets has previously been described in [17], and is summarized below. The experiment described was performed under deep anesthesia using procedures approved by the Institutional Animal Care and Use Committee of the University of Utah and conformed to the Guide for the Care and Use of Laboratory Animals. An excised canine heart was Langendorff-perfused using a mixture of whole blood and Tyrode’s solution. Arterial blood was supplied from a second canine under deep anesthesia. A right ventricular cannula extracted venous blood from the isolated heart and returned it to the support dog via a blood pump and a cannula in the jugular vein. The isolated, perfused heart was suspended in a human torso shaped tank with 192 electrodes (with inter-electrode spacing 40.2 ± 16.8mm) and filled with an electrolytic solution (500 Ω-cm). The heart was instrumented with 33 intramural plunge electrode needles. In addition, ventricular signals were sampled using a 247-electrode epicardial sock (inter-electrode spacing 6.5 ± 1.3 mm). Intramural needles were used to stimulate the heart using bipolar stimulation from the RV. The epicardial, intramural, and torso tank electrodes were referenced to a Wilson’s Central Terminal and were sampled at 1000 Hz simultaneously for five seconds during RV pacing. At the end of the experiment, a three-dimensional mechanical digitizer (Microscribe from Immersion Corp.) was used to locate landmarks marked on the tank and electrode array, which then provided the reference points for proper orientation of the sock geometry within the torso tank.

## III. ECG SIGNAL PROCESSING METHODS

**Fig. 2.**
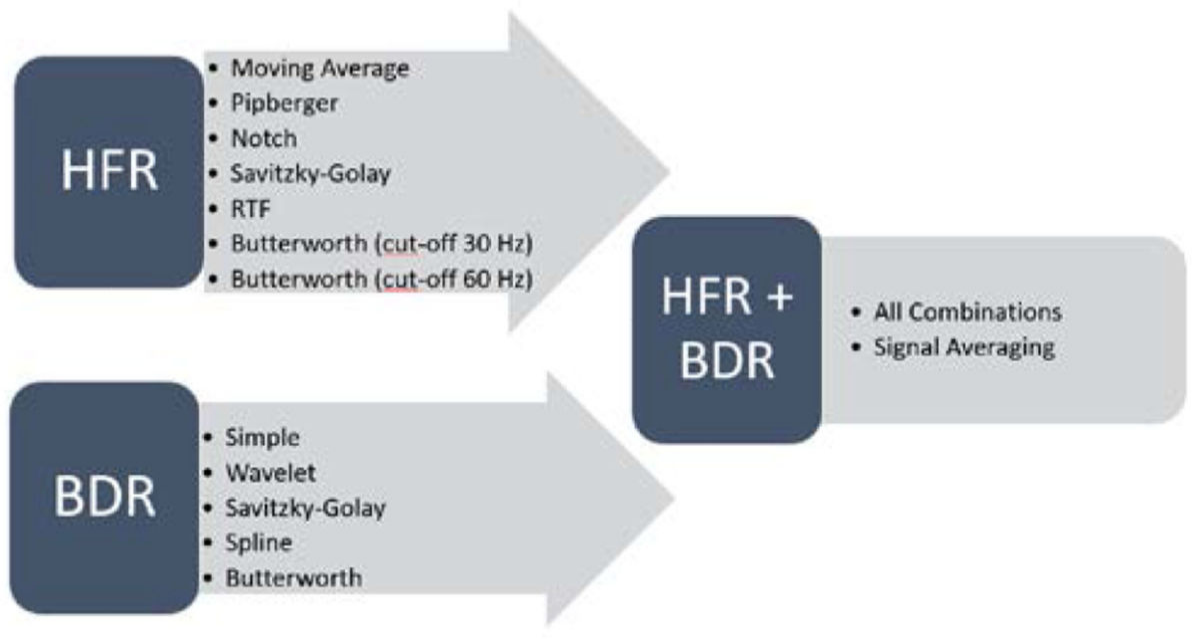
Filters applied to each signal included those for high frequency removal (HFR), baseline drift removal (BDR) and all combinations of the two types of filters. In addition, signal averaging was used as a special HFR + BDR filter.

Torso signal processing methods and their associated parameters were collected from different international research centers working with ECGI. These were divided into three distinct categories (Fig. 2). The first were methods for high-frequency noise removal (HFR) including the 50 or 60 Hz power line interference. The second were methods for low frequency baseline drift removal (BDR) alone. The final was for methods that combined HFR and BDR. For this final category, all HFR and BDR methods were combined. Furthermore, a signal averaging method was included in the study. The different filtering categories resulted in a total of 49 different processed signals including the original raw potentials. BDR1 was applied to directly recorded signals for all data sets. The different filters used are outlined below.

### A. High Frequency Noise Removal (HFR)

Seven different HFR methods were applied:

### 1) Moving Average Filter (HFR1):

A simple moving average filter computed over the 20 or 17 ms time window, corresponding to one cycle of the line noise present in each dataset (50 Hz for Bordeaux, 60 Hz for Utah). The weight function is constant normalized by the number of samples in window.

#### 2) Pipberger’s Filter (HFR2):

Also a moving average filter, suggested in [18], where the averaging window corresponds with the length of two cycles of 50 or 60 Hz (40 or 33 ms), and the summed samples are weighted by a cosine function.

#### 3) Notch Filter (HFR3):

Signals were transformed into the Fourier space, and a notch filter was applied around the line frequency and its harmonics before applying the inverse Fourier transform.

#### 4) Savitzky-Golay Filter (HFR4):

A Savitzky-Golay FIR smoothing filter was applied to the data using the ‘sgolayfilt’ function in MATLAB R2018a with an order of 3 and a frame length of 20 or 17 ms.

#### 5) Rational Transfer Function (HFR5):

A rational transfer function (RTF) implemented using MATLAB, the default filter implemented in the open-source PFEIFER toolbox [19]. This filter serves as a weighted running average with an 11-element kernel size.

#### 6) Butterworth Low Pass Filters (HFR6 and HFR7):

Two different IIR low pass filters with filter order 7 at cut-off frequencies, 30 Hz (HFR6) and 60 Hz (HFR7) to be below or at/above the line frequency noise present in each dataset [20].

### B. Baseline Drift Removal (BDR)

Five methods for BDR were applied on the original signal:

#### 1) Simple (BDR1):

A naive baseline removal approach in which the mean over a 20 or 17 ms time window during the isoelectric period prior to the QRS was subtracted from each signal.

#### 2) Wavelet-Based (BDR2):

A wavelet bandpass filter (0.5–150 Hz) was applied with 20 levels of decomposition performed using Coiflet wavelets with four vanishing moments.

#### 3) Savitzy-Golay (BDR3):

A Savitzky-Golay FIR smoothing filter was applied to the data using the ‘sgolayfilt’ function in MATLAB with a polynomial order of 3 and a frame length of 3000 ms.

#### 4) Cubic-Spline (BDR4):

Predefined expected isoelectric points of the measured signal were used for computation of the zero line by fitting the isoelectric line with a cubic function.

#### 5) Butterworth High Pass Filter (BDR5):

IIR high pass filters with filter order 5 at cut-off frequency of 0.5 Hz.

### C. High Frequency Noise and Baseline Drift Removal

The methods for HFR and BDR were combined, by first performing each of the seven HFR on the raw signal, then applying the five BDR to each of these seven signals. This resulted in a total of 35 different HFR + BDR combinations.

### D. Signal Averaging (SA)

In addition to simple HFR and BDR methods, a signal averaging method was implemented [21]. Briefly, baseline wander was first removed using the wavelet BDR method (BDR2). The time window containing one heart beat was defined by hand as the QRST interval. The tank signals were then decomposed using a principal component analysis (PCA). The first principal component was defined as the virtual lead and the pre-defined heart beat in this virtual lead as the virtual template. The virtual template was compared with each beat of the virtual lead, by cross correlation. The position for the alignment was determined as the position where the cross correlation was maximal. Finally beat averaging was performed over all recorded and aligned beats for each lead.

## IV. INVERSE MAPPING METHODS

Tank signals which were absent or of poor quality were excluded from the computations (3 ± 2 channels removed). The ECGI approach assessed in this study was chosen to reflect the most common approach used by the different research centers.

### A. Problem Definition

The electrograms on the epicardial nodes are linearly related to torso measurements:

(1) y(t)=Ax(t)+n(t)

where $x(t)\in {\mathbb{R}}^{N\times T}$ and $y(t)\in {\mathbb{R}}^{M\times T}$ are the epicardial potential and the torso measurement vectors at time *t*, respectively, $A\in {\mathbb{R}}^{M\times N}$ is the forward matrix, and $n(t)\in {\mathbb{R}}^{M\times T}$ is the vector representing noise in the measurements.

### B. Forward Problem Solution

The boundary element method (BEM) was used to define the forward matrix, employing a homogeneous conductivity between a refined epicardial mesh (internode spacing 5.1±2.1 mm for Bordeaux and 7.4 ± 2.0 mm for Utah data) and a refined tank mesh (internode spacing of 14.4 ± 5.9 mm for Bordeaux and 24.2 ± 5.1 mm for Utah data). After the forward matrix is obtained for these refined meshes, the rows corresponding to torso measurement electrodes were sampled to form a reduced forward matrix, which we denote as **A** in (1), relating the electrograms (in the refined epicardial meshes) to torso measurements.

### C. Inverse Problem Solution

Inverse solutions were found by applying the Tikhonov regularization method [22] to the problem in (1) at each time instant separately. Thus, time index was removed from the following description of the method. This method aims to achieve a trade-off between a good fit to the measured data and an a priori constraint on the solution, thus minimizing the cost function:

(2) $$J(\text{x})=∥\text{Ax}-\text{y}{∥}^{2}+\lambda^{2}∥R\text{x}{∥}^{2}$$

with respect to **x** at each time instant, where∥⋅∥ is the L2-norm, **R** is a regularization matrix representing the constraint on the solution and λ is a regularization parameter controlling the trade-off between the two components of the cost function. In this study, **R** was chosen as the identity matrix (zero-order regularization). Per C. Hansen’s L-curve method [23] was applied λ(*t*) at each time instant. The median over time was computed and defined as the final λ value that was used to solve the inverse problem at each time instant.

## V. EVALUATION METHODS

First, the lambda value used for each inverse solution and the processed torso signals were evaluated using two metrics:

1. *Signal-to-Noise Ratio for High Frequencies (SNR-HF):* The RMS voltage (RMSV) was computed across all leads after a simple baseline correction (BDR1). The SNR-HF was then taken as the mean ratio between the QRS and noise amplitudes during an isoelectric period 40 or 34 ms prior to the QRS for Bordeaux and Utah data respectively.
2. *Baseline Shift:* was defined as the deviation of the isoelectric point prior to the QRS. To account for noise, the average was taken over 20 or 17 ms. The mean absolute deviation over all leads was used.

The inverse solution was found for each beat in the signal (14–31 beats) and compared to the same beat measured by the sock. As SA produced a single beat, reconstructed electrograms were compared to all un-averaged sock beats. The inverse solutions were obtained for refined epicardial meshes and comparisons were carried out at a subset of the nodes corresponding to sock measurement locations (108 and 247 leads for Bordeaux and Utah data respectively).

The following features of the recorded and ECGI reconstructed electrograms were quantified and compared:

1. Electrogram Amplitude: Electrogram amplitude was measured as the mean of the peak-to-peak amplitudes from each lead. Comparison of ECGI to recorded electrogram amplitudes were made using the absolute difference and the relative difference using the maximum recorded amplitude for normalization.
2. Electrogram Morphology: The morphology of ECGI reconstructed electrograms were compared to those recorded over the QRS using a Pearson’s correlation).

Finally, activation times markers were defined from sock electrograms as the time of minimum derivative (dV/dt). A spatio-temporal algorithm was used to define activation times from ECGI electrograms [24]. Activation maps were compared using the following metrics

1. Activation Wavefront: was compared using the Pearson’s correlation and the mean absolute error (MAE).
2. Pacing site Localization Error (LE) - The definition of the pacing site was automated and defined as the site with the earliest activation time where the median of all neighboring points activated within 30 ms (to prevent selecting a site within a misplaced activation marker). If multiple points demonstrated the same activation time, the mean of these points was taken as the earliest activation. The LE was then computed as the Euclidian distance between the ECGI and recorded pacing sites.

Statistical analysis was conducted using GraphPad Prism 7.04. For each metric the significance of differences was tested using 1-way ANOVA with p < 0.05 defined as significant. A repeated measures method was used for comparisons within a data set and unpaired for comparisons between the two data sets. Data are expressed as mean ± SD unless otherwise stated. Figures contain representative data, with Tables containing the complete results available as an online supplement.

## VI. RESULTS

### A. Torso Surface Potentials and Lambda Values

**Fig. 3.**
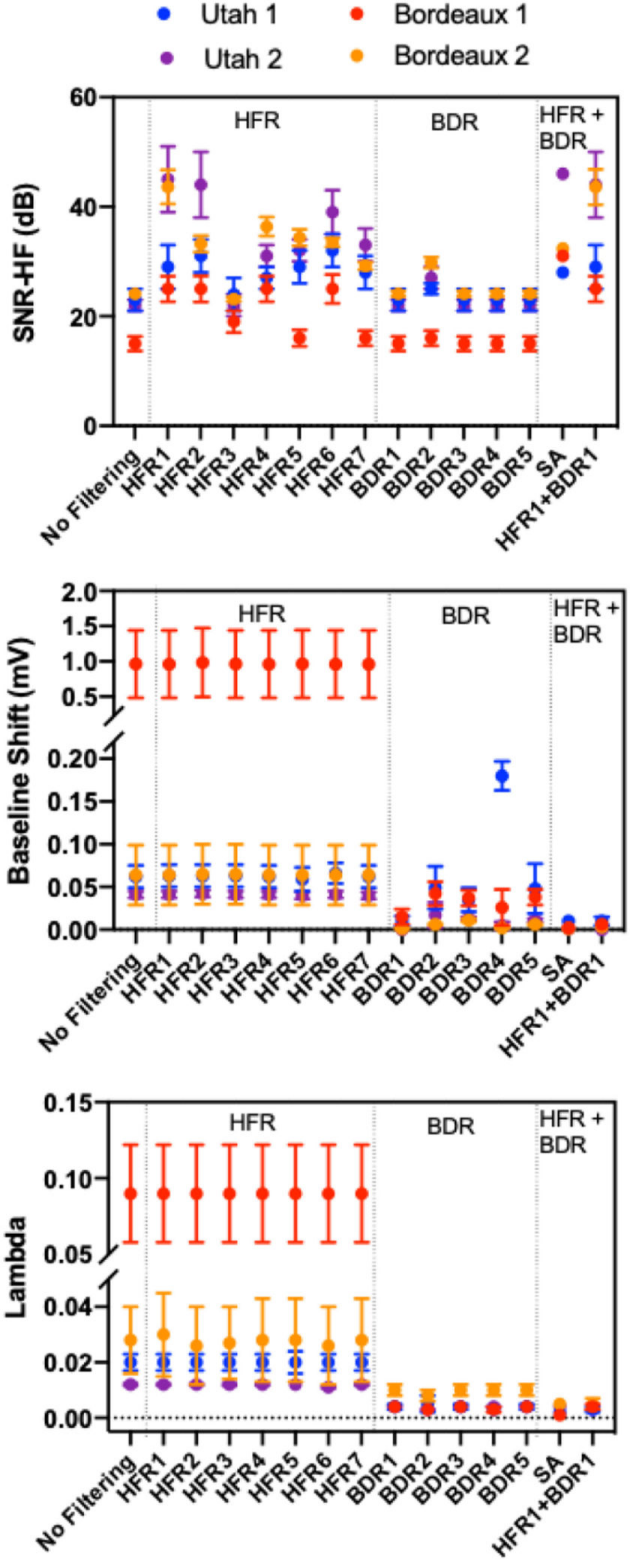
Body Surface ECG noise properties and Lambda values for each data set. Results presented for selected high frequency removal (HFR), baseline drift removal (BDR) and combinations of the two methods. In addition, signal averaging (SA) was used as a special HFR +BDR filter.

SNR-HF, Baseline Shift and lambda values for selected processing methods for all data sets are presented in Fig. 3. As expected, the SNR-HF of the raw potentials for the three clean data sets were significantly larger than for the noisy data (Bordeaux 1; red), but also had significantly less baseline shift and 5x smaller lambda values.

All HFR methods except the notch filter (HFR3) significantly improved the SNR-HF compared to no filtering for all data sets (p < 0.0001) with the moving average (HFR1), Pipberger (HFR2) and the 30 Hz Lowpass filter (HFR6) producing the best results. The improvements seen with the RTF (HFR5) and a low-pass filter above the line noise (HFR7) were small. The Notch filter significantly improved SNR-HF for Bordeaux 1 where lines noise was prominent. HFR did not significantly change the baseline shift for any data sets. While there was a significant difference in lambda values between HFR methods, the absolute differences were minimal.

For all data sets, no BDR methods significantly improved the SNR-HF of the torso signals except the wavelet method (BDR2) as this includes a high frequency cut-off of 150 Hz. Though significant, the improvement was small. Most BDR methods demonstrated a marked improvement in the baseline shift. However, for the Utah 1 data set (blue), the wavelet (BDR2) and the high pass filter (BDR5) did not significantly change the baseline offset. Furthermore, the spline method (BDR4) significantly increased the baseline shift. Here, while the spline fit was accurate, it created a constant offset across the entire signal due to the automated method for finding the isoelectric points defining a point at the start of the QRS. For all data sets, there was a significant reduction in lambda with BDR, with no difference between the BDR methods.

For all data sets, there was no significant improvement or reduction in SNR-HF in most cases with the addition of BDR to HFR compared to using HFR methods alone (Hence only HFR1+BDR1 is represented). The exceptions to this are with the Notch filter which in combination with the Wavelet BDR (HFR3+BDR2) improved SNR-HF by 3–10 dB for the different data sets due to the added high-pass filtering effects of the wavelet filter (p < 0.001). There was also no further substantial reduction in baseline shift with the addition of HFR to BDR compared to BDR alone. The lambda value was significantly reduced in the majority of cases. However, as with the Baseline shift and SNR-HF the relative change was minimal with a mean difference ranging from 0.00001 to 0.0008.

SA improved the SNR-HF and baseline shift compared to no filtering for all data, as well as reducing lambda values (p < 0.05). Globally, SA showed similar SNR-HF and baseline shift values to many HFR + BDR combinations, though the SNR-HF was greater than any method for Bordeaux 1 data.

### B. Electrogram Reconstruction

**Fig. 4.**
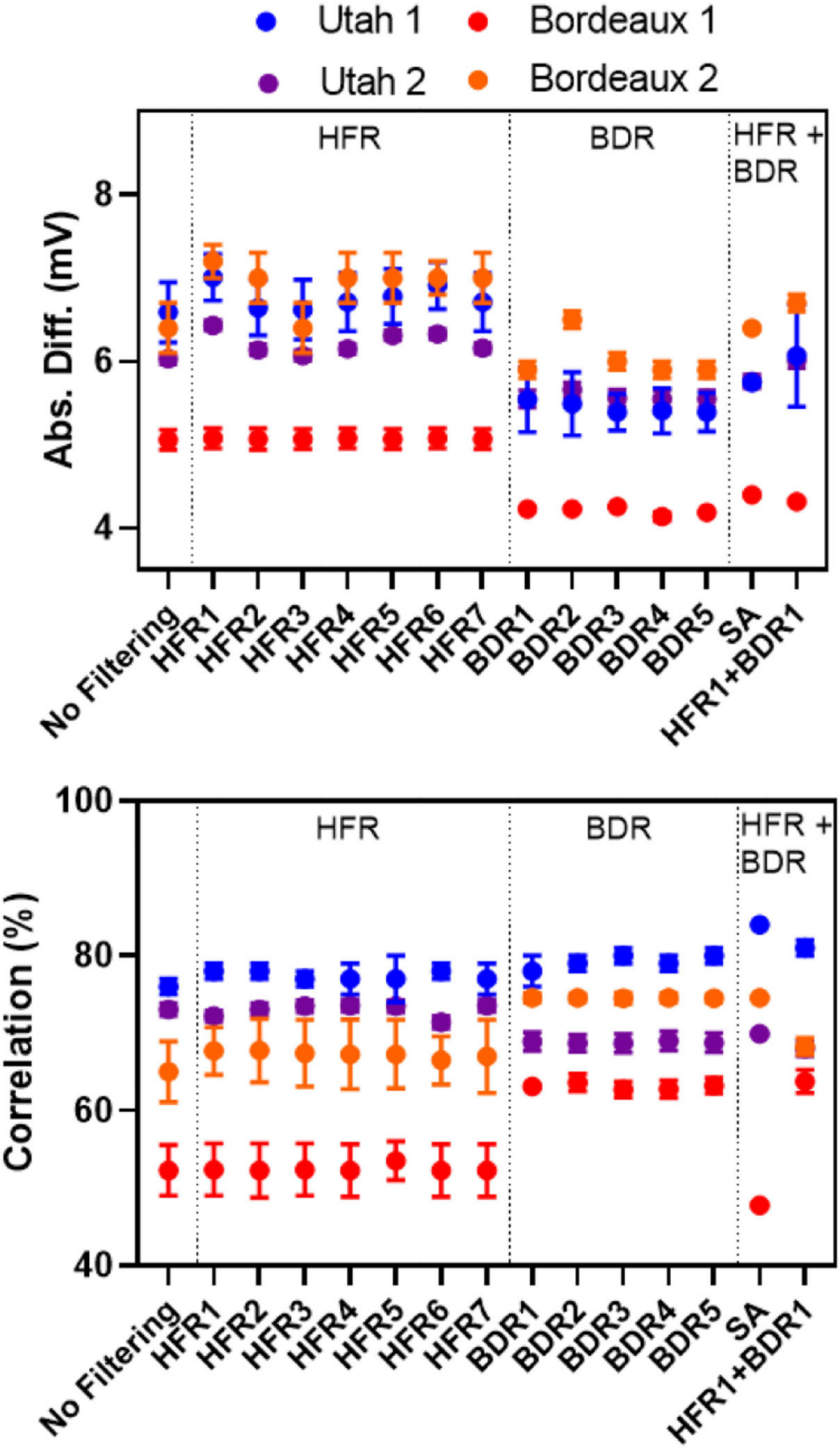
Comparison between recorded and ECGI electrograms using absolute difference and correlation. Results presented for selected HFR, BDR and their combination. In addition, SA was used as a special HFR + BDR filter.

Fig. 4 presents the absolute amplitude difference and correlation between ECGI and recorded electrogram over the QRS interval for selected ECG processing techniques.

With no filtering of the ECG, ECGI electrogram amplitudes were smaller than recorded for all data sets. With no filtering, the Noisy data set showed significantly worse median correlation in electrograms QRS waveform between reconstructed and recorded potentials than any Clean data set (52 ± 3% vs. 67± 4 to 76 ± 1%).

**Fig. 5.**
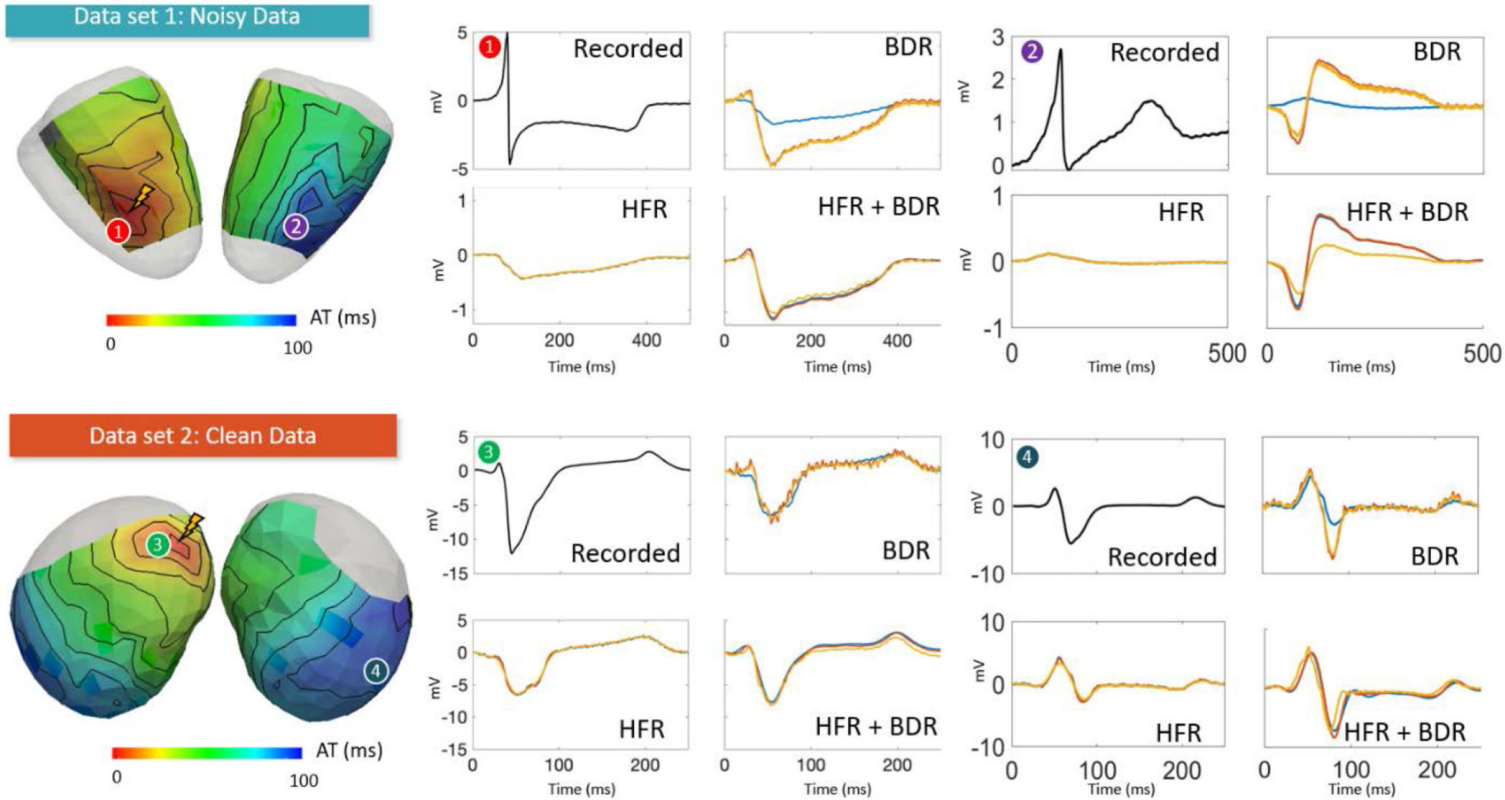
Representative recorded and reconstructed electrograms using selected signal processing methods. Representative recorded EGMs for both data sets at two electrode locations are marked on recorded activation maps (left). HFR, BDR and HFR+BDR depict the selected reconstructed EGMs for HFR only, BDR and the combination of HFR+BDR respectively. Within the HFR plots, the blue line represents reconstructions with no filtering, the yellow line with a moving average filter (HFR1) and the orange line with a notch filter (HFR3). Within the BDR plots, the blue line represents reconstructions with no filtering, the orange line with a wavelet filter (BDR2) and the yellow line with a simple BDR (BDR1). Within the HFR+BDR plots, reconstructions after a low pass filter (HFR6; blue) and the RTF filter (HFR5; orange) combined with the spline filter (BDR4) are presented alongside the signal averaged reconstruction (yellow).

HFR did not significantly improve electrogram amplitudes compared to no filtering for the Noisy data. For the Clean data sets all methods significantly decreased amplitudes except the notch filter (HFR3). The moving average (HFR1) showed the largest difference. This can be seen in Fig. 5, which presents representative recorded (black) and ECGI electrograms for the Bordeaux 1 (top) and Utah 1 (bottom) data sets at two electrode locations marked on recorded activation maps (left). Here, the ECGI electrograms using the moving average (HFR1) alone (yellow) are slightly smaller than those without filtering (blue) or using a notch filter (HFR3; orange) in the clean data.

**Fig. 6.**
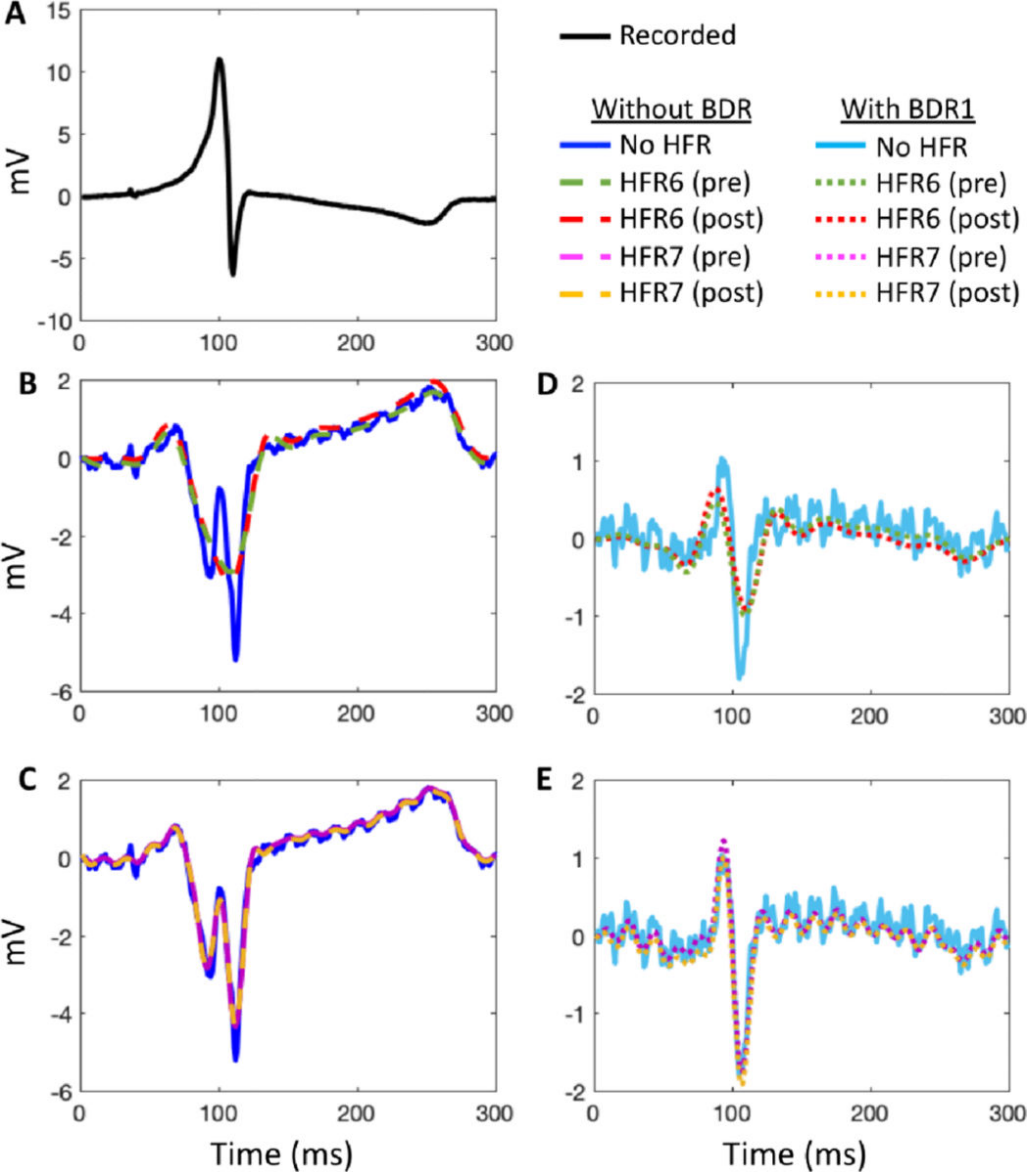
Example (A) recorded and (B,C,D,E) reconstructed electrograms with (right) and without (left) simple BDR (BDR1), and either no HFR, a 30 Hz (HFR6) or a 60 Hz (HFR7) low pass filter used either pre-or post- reconstruction.

While certain HFR methods did significantly improve the correlation for both Bordeaux 1 and Utah 1 data compared to no filtering, the improvements were minimal (0.2–2.3% maximum). Visual inspection demonstrated no obvious changes in QRS morphology when correlation was improved other than a reduction in high frequency noise content (Fig. 5 - HFR). For the Utah 2 data set, the moving average filter (HFR1) and the 30 Hz low pass filter (HFR6) significantly reduced the correlation values. For this data set, visual inspection demonstrated that these filters have resulted in an over-smoothed reconstruction on a small area of the heart. This is demonstrated in Fig. 6A and B with a recorded electrogram (black) and the equivalent inverse reconstruction without filtering (dark blue) and with a 30 Hz low pass filter (HFR6; green dashed). As can be seen, HFR6 has over-smoothed the inverse reconstruction, removing the second downstroke that corresponds to the intrinsic reflection. By using a higher 60 Hz cut-off for the low-pass filter (Fig. 6C magenta dashed); the second down stroke is preserved. Interestingly, by performing HFR post-reconstruction (Fig. 6B red and C yellow lines), we produce the same signal as when filtering was performed pre-reconstruction. This was also seen for reconstructions after BDR (Fig. 6D and E), where HFR7 and 8 performed pre-reconstruction (green and magenta dotted respectively) match the electrograms with HFR7 and 9 performed post-reconstruction (red and yellow dotted respectively).

BDR improved potential amplitudes for all data compared to no filtering with a significant reduction in absolute differences (Fig. 4). The spline method (BDR4) provided the greatest improvement, and simple/wavelet methods (BDR1/BDR2) the least. While amplitudes were improved, the noise level in the electrograms was also dramatically increased reducing the SNR, as seen in Fig. 5 BDR electrograms for both data sets, with no filtering in blue, wavelet (BDR2) in orange and simple (BDR1) in yellow. The wavelet method provided some filtering of high frequency noise as expected from body surface signals. BDR significantly improved the median correlation between ECGI and recorded potentials compared to no filtering for both Bordeaux and the Utah 1 data. However, for the Utah 2 data the correlation was overall reduced. There were no significant differences between the BDR methods for all data sets (p > 0.99).

In all data sets, when improvements in QRS morphology occurred, these were substantial. As seen in Fig. 5 BDR plots key electrogram features were better reproduced in ECGI electrograms using the wavelet (orange) and simple (yellow) BDR compared to no filtering (blue); including the initial R-wave in electrode 1 and 3, and the S-wave in electrode 4. This is also seen in Fig. 6 for the Utah 2, where the R-wave is not present in reconstructions without BDR (Fig. 6B and C), but is present in all reconstructions with BDR (Fig. 6D and E). In a few electrograms, detrimental changes were found after BDR, most prominently in the Utah 2, e.g., in Fig. 5 electrode 2 an initial downstroke is reconstructed after BDR that does not exist in the recording nor in unfiltered or HFR-alone reconstructions.

Unlike with HFR, changes in electrogram morphology with BDR were only produced when filtering was applied prior to inverse reconstruction, and could not be reproduced through filtering post-reconstruction. The combination of HFR and BDR on ECG signals provided a significant improvement in the signal amplitudes for all datasets by reducing the absolute amplitude difference when compared to HFR alone (p < 0.05), but were increased slightly compared to BDR alone. For both Bordeaux and Utah 1 data sets, most combinations of HFR methods with BDR also significantly improved the correlation of QRS EGMs compared to BDR alone though these absolute changes were minimal (<0.04 change in the median correlation). Only in combination with the RTF filter (HFR5) were correlation values reduced compared to BDR alone (p < 0.05). Representative reconstructed electrograms in Fig. 5 HFR+BDR demonstrate that these changes are limited to a reduction in high frequency noise content, with reconstruction using a low pass filter (HFR6) (blue) and the RTF filter (HFR5) (orange) combined with spline BDR (BDR4) presented alongside the signal averaged reconstruction (yellow).

Signal averaging, like BDR and BDR + HFR, improved signal amplitudes compared to no filtering. While for the Utah 1 and Bordeaux 2 data sets, correlation of QRS EGMs were significantly better than for any other method, for the noisy Bordeaux 1 data set and the Utah 2 data set they were significantly worse. This can be seen in Fig. 5 electrode 2, where the signal averaged reconstruction (yellow plot HFR + BDR) has inverted the electrograms completely now showing no similarity to the recorded plot. For all data sets, signal averaged reconstructions still contain high frequency noise likely because not enough beats are used in the averaging (14 to 31 beats used).

### C. Activation Maps

**Fig. 7.**
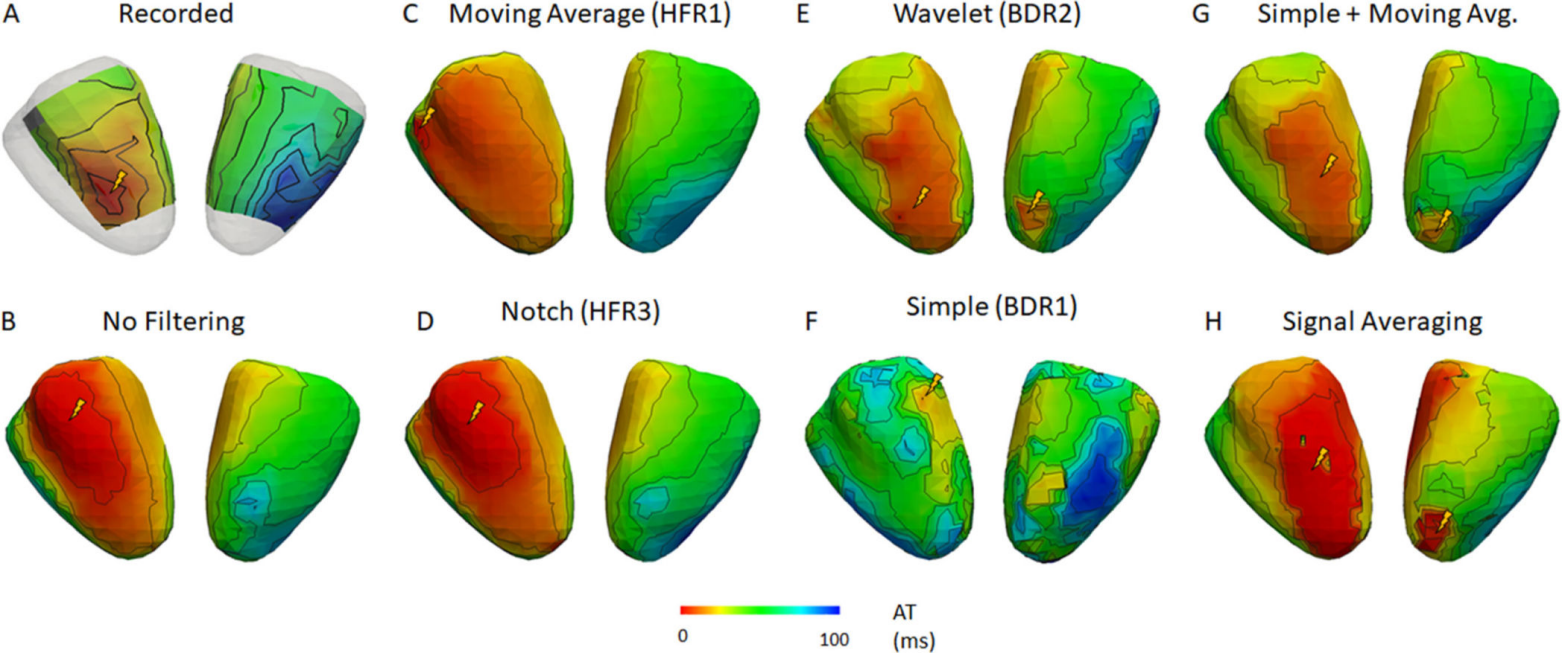
Representative recorded and reconstructed activation maps using selected signal processing methods for the Bordeaux 1 data set. Activation maps show right (left) and left (right) ventricular views of the heart. Lightning bolt represents sites of earliest activation as defined by each map.

**Fig. 8.**
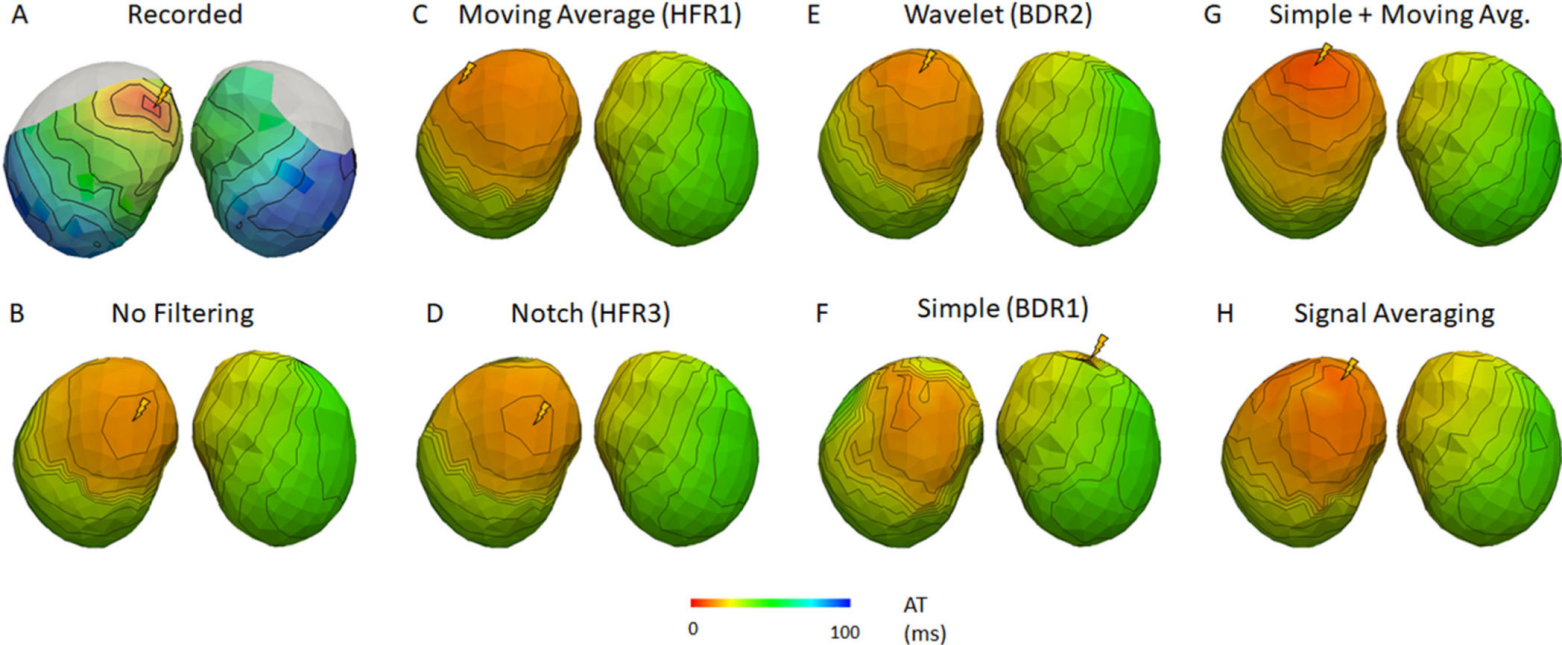
Representative recorded and reconstructed activation maps using selected signal processing methods for the Utah 1 data set. Activations show and anterior (right) and posterior (left) view of the heart. Lightning bolt represents sites of earliest activation as defined by each map.

**Fig. 9.**
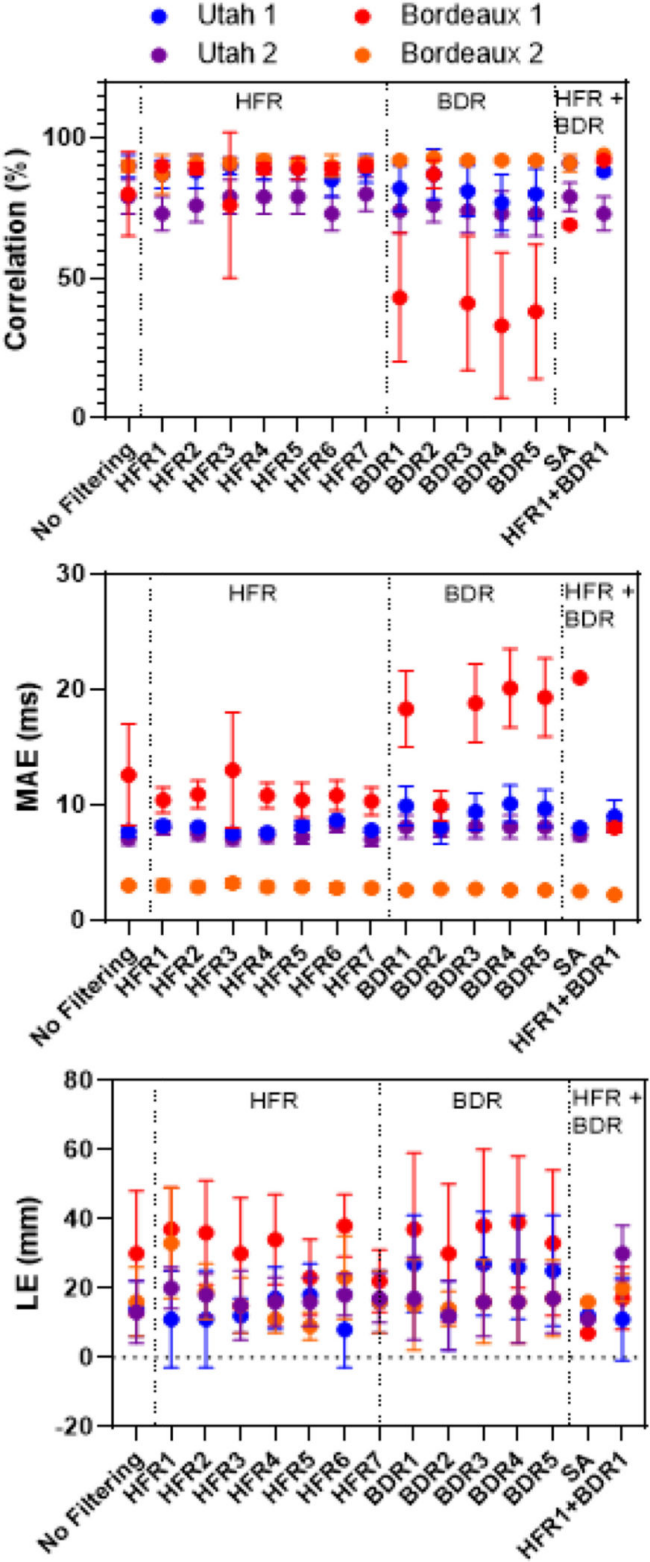
Comparison between recorded and reconstructed activation maps using correlation, mean absolute error (MAE) and localization error (LE). Results presented for selected HFR, BDR and combinations of the two methods. In addition, SA was used.

Figs. 7 and 8 present representative recorded (A) and ECGI activation maps for the Bordeaux 1 and Utah 1 data sets, respectively with (B) no filtering, (C and D) HFR alone, (E and F) BDR alone, (G) BDR+HFR and (H) signal averaging. Fig. 9 presents the correlation and mean absolute error (MAE) between ECGI and recorded activation times as well as the localization error (LE) for selected processing methods.

**Fig. 10.**
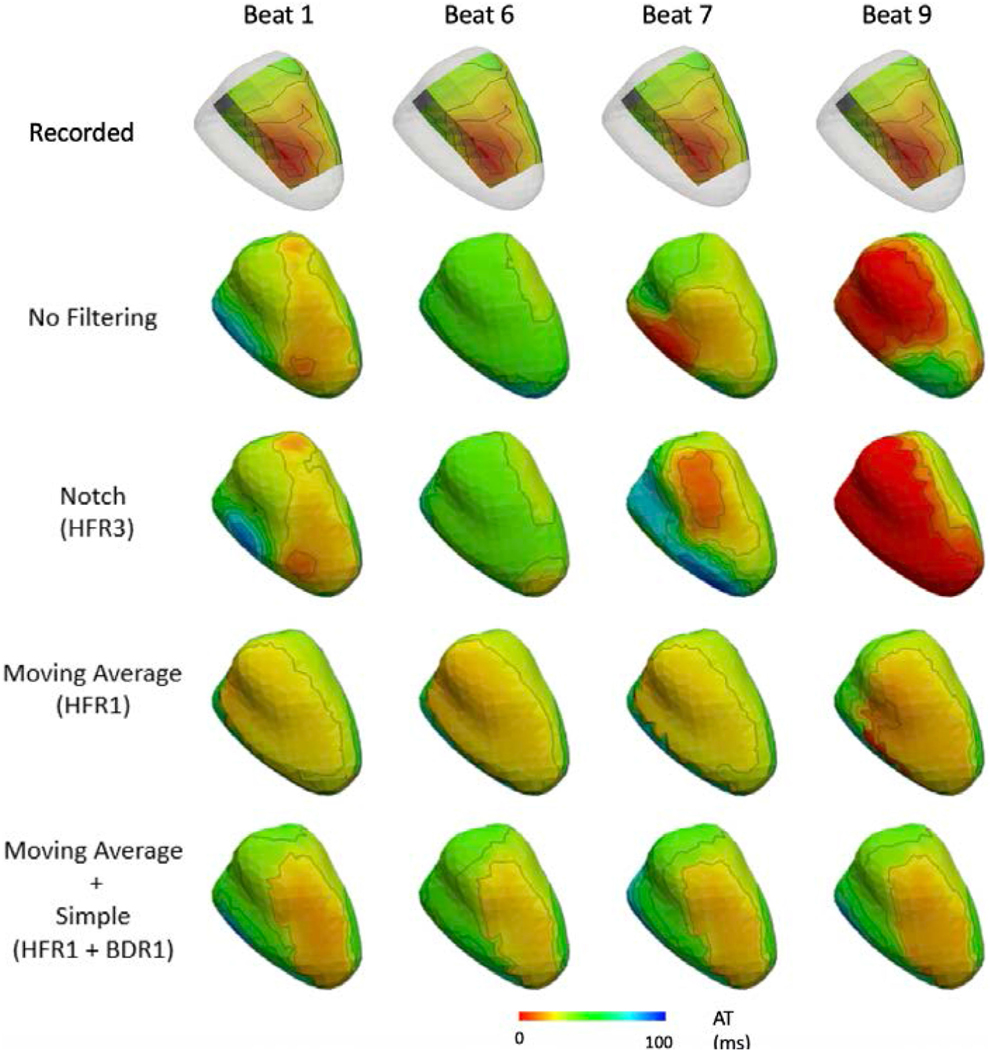
ECGI activation maps for Bordeaux 1 data demonstrating the beat-to-beat variability (left-right) using selected processing methods (top-bottom).

Activation maps without filtering were well correlated with those recorded and had a low MAE for all data, though results were best for the clean data sets. Pacing site LE was also significantly higher for the noisy Bordeaux 1 data set (p < 0.05). Variation between the activation maps on a beat-to-beat basis was also greater for the noisy Bordeaux 1 data as demonstrated by the larger standard deviation in correlation and MAE compared to the other data. These beat-to-beat changes in the ECGI activation maps are presented in Fig. 10 for the Bordeaux 1 data. Beat-to-beat variability in the recorded sock data was minimal, with each activation maps showing no discernible difference, illustrating this variability arises when significant noise is present in the ECG signals.

For the Bordeaux 1 data, HFR significantly improved correlation of activation maps with those recorded, except with the Notch filter. For the other data sets, no methods changed the correlation except the moving average (HFR1) and 30 Hz low pass (HFR6), which reduced it. Closer inspection of reconstructed maps from the Bordeaux 1 data set revealed that filtering removed isolated activation marker errors (HFR3; Figs. 7 right wall and 8 posterior wall). For the majority of the beats, the Notch filter showed similar but smoother activation maps than no filtering. However, in approximately a ¼ of the beats the activation map was changed drastically in the Noisy data, as seen in Fig. 10 beat 7. Inspection of the electrograms in altered regions showed the global QRS morphology was unchanged (as was noted in the previous section), but large amplitude high frequency noise was still present after the notch filter that has likely altered activation marker placements. Other HFR methods also altered the activation sequences compared to no filtering (example Fig. 7 with the moving average filter) but unlike with the notch they showed little beat-to-beat variability. On the other hand, for the other data sets there were no substantial visual differences between the HFR filtered and the unfiltered reconstructed activation maps other than shifts in the early activated region (Fig. 8B, C and D). Like with the Bordeaux 1 data, this was not reflected in the electrograms which had very similar morphologies in the early activated region. The already minimal beat-to-beat variability also did not change with filtering. For both Bordeaux and Utah 1 data sets, while there were HFR methods that improved the mean LE, or reduced the standard deviation, none were significant. For Utah 2, LEs were larger but not significantly. Interestingly there were methods that improved LE for one data set but deteriorated results for another. The only consistent change was for the moving average filter (HFR1) that produced larger LE than using no filter (p < 0.05).

After BDR, the increased noise amplitude, seen in Fig. 5, negatively impacted activation marker placement, as seen in Figs. 7 and 8 where activation maps became patchy after BDR. Overall there was a decrease in activation accuracy with BDR despite the improvements seen in electrogram accuracy. The wavelet method (BDR2) was an exception to this, improving activation map accuracy in terms of correlation and MAE as it provided some HFR to electrograms. However, for the Bordeaux 1 data set, an artefactual second region of early activation appeared on the posterior wall impacting LE for all activation maps after BDR (Fig. 7E). Inspecting the electrograms in this region revealed an artefactual early downslope that is reconstructed only after BDR (as demonstrated in Fig. 5 electrogram 2). For all except the Bordeaux 2 data, all of the BDR methods except the wavelet method increased the mean and standard deviation of the LE for the earliest site of activation. For the Utah 1 data set this increase was significant (p < 0.05).

The combination of HFR and BDR provided no substantial further improvement in correlation or MAE over HFR alone. Furthermore, the pattern of activation was not substantially changed. Inspection of the maps themselves demonstrated that the artefactual region of early activation was present in all HFR+BDR reconstructions for the Bordeaux 1 data set (example Fig. 7G). No such artefacts were seen in the other data sets. Though not significant, the mean and SD in LE improved substantially for most combinations, particularly for cases that had increased with only HFR (HFR1, 6).

SA produced activation maps with similar correlation and MAE values to no filtering for the Utah and Bordeaux 2 data but lower correlation and higher MAE for the Bordeaux 1 data. While activation maps for the Utah data looked very similar to those without filtering, for the Bordeaux 1 data there were several artefacts including a second site of activation on the right surface (like with all BDR or BDR+HFR filters). For both all data, the SA found the earliest site of activation <15 mm, in the closer range compared to any other processing method.

## VII. DISCUSSION

This study has demonstrated the impact of different signal processing methods on an epicardial potential based inverse method using four data sets from two distinct experimental setups. As there was no one optimal processing method for all data, we recommend signal processing be applied on a case-by-case basis. Several trends were applicable to all data sets that can help guide the choice of processing method used and ensure the best inverse solution is found:

1. HFR does not impact electrogram reconstruction accuracy, but does impact post-processing to extract features such as activation times. HFR should be applied post-reconstruction to ensure over-filtering does not occur. The HFR methods and parameters will depend on the features one wishes to extract.
2. BDR improves reconstructed electrogram due to a reduction in lambda value selected. We recommend using the simple method (BDR1) as this is sufficient to see these improvements.
3. Signal averaging may be a useful processing tool but care should be taken in aligning the beats.

### A. High Frequency and Baseline Drift Removal

Torso signals with baseline drift present (i.e. no filtering or HFR) resulted in a higher regularization parameter choice using the L-curve method (Fig. 3). The more baseline drift present, the more regularization the signals received. With Tikhonov zero-order regularization, the presence of baseline drift resulted in very low amplitude and smooth reconstructed signals. Conversely, reconstructions after BDR improved electrogram morphology and amplitudes.

Improvement in electrograms after BDR included the appearance of key features like the initial R-wave (Fig. 5 and 6). Though less common, detrimental changes could also be found, such as the development of a double intrinsic deflection (Fig. 5). Both positive and negative changes were present with all BDR methods including the simple method (BDR1), meaning these changes are likely due to the selected lambda value rather than distortion in the torso surface QRS from filtering. To verify this, reconstructions were compared before and after BDR using a fixed lambda value and demonstrated no perceivable difference in QRS morphology. We conclude the changes in electrogram morphology after BDR are due to the large reduction in lambda value selected. This conclusion is further supported by HFR-only reconstructions where electrogram morphologies and lambda values are not significantly different from those with no filtering. As positive changes are also seen with the simple method, we would advise this approach as there appears to be no benefit from using more complex BDR approaches. In order to limit detrimental changes, the method for computing the lambda value needs to be optimized.

While BDR significantly improved specific morphological features, the presence of high frequency noise had the biggest impact on activation maps, and therefore HFR methods were required to improve their accuracy. If the reconstructed signals had little high frequency noise, HFR had little impact (i.e. the three clean data sets after only HFR). Of the HFR methods evaluated in this study, the notch filter was the only method deemed insufficient to improve activation time mapping given the high frequency noise was still present in the signal. Even a low pass filter above the line noise improved activation reconstruction accuracy, indicating the line noise does not impact activation marker placement.

The moving average (HFR2) and 30 Hz low pass (HFR6) filter methods were detrimental to defining activation times for the Utah 2 data set by over-smoothing the reconstructed electrogram (Fig. 6). Interestingly, while the global activation pattern was improved with the moving average filter for the other data sets, the LE of the pacing site was larger than without filtering. We suspect this is due to over-smoothing the early QRS at the body surface, that resulted in an increase in the presence of isoelectric-line clustering or apparent line of conduction slowing and shifted the early activation site to these borders, as seen in Figs. 7 and 8 (earliest activation denoted with yellow lightning bolt). By using a smaller time window for averaging this defect may not occur.

Further investigation into filtering artefacts at the body surface demonstrated that if these are present they will remain in reconstructions. For example, the low pass filter of 30 Hz created an obvious ringing on either side of QRS in the Clean data set. Whilst this does not affect the correlation values or timing of the intrinsic deflection, the ringing was still present in the reconstructed electrograms and could be mistaken as late potentials after the QRS.

For a purely spatial (static) inverse approach, as used in this study, the regularized pseudo-inverse matrix is a purely spatial operator, treating every timestep of the torso signals individually. A linear temporal filter can be expressed as a matrix M, likewise treating every timestep of the torso signals individually

$$y{\left(t\right)}_{filtered}=My(t)$$

Therefore, the order of temporal filtering and purely spatial (static) reconstruction can be also formally be exchanged, as long as the same lambda is used. We therefore recommend applying HFR after reconstruction when using a purely spatial (static) inverse approach to ensure there are no deformations in morphology and accuracy in activation map computation. However, it should be kept in mind that for spatio-temporal inverse approaches the order does make a difference.

### B. Signal Averaging

The application of averaging on an ECG makes three assumptions, i) the ECG pattern repeats cyclically, ii) the high frequency noise superimposed on the signal is a random noise with zero mean value, and iii) ECG can be correctly aligned to perform averaging. If all three assumptions are correct, then signal averaging will prevent any distortion of the ECG and yield a considerable decrease of the noise dispersion. Here we have found signal averaging to be beneficial for the clean data and detrimental for the noisier Bordeaux 1 data set. This is despite the SNR-HF being improved and QRS waveforms at the tank surface appearing normal. We assume the difference in results comes down to the alignment of the beats; good for the clean data sets with no QRS deformation at the body surface and bad for the noisy data set resulting in an unperceivable QRS deformation in the ECG. It is clear that if alignment is not good, signal averaging can be more detrimental than any other filtering approach to the inverse solution. This is confirmed with the large deformations seen in the reconstructed electrograms for the Bordeaux 1 data with signal averaging that are not present for the other data sets. We hypothesize that if the optimal alignment approach can be determined, it would provide the best filtering tool to use for stable rhythms as there would be no QRS deformation. As such we have commenced an investigation into different signal averaging methods in relation to the inverse problem.

### C. Limitations

The results presented should be considered in light of limitations. First, while a large variety of signal processing methods have been used, the study has only investigated one inverse problem “pipeline”. That is, one forward model (BEM), one regularization technique (zero-order Tikhonov), one method to define lambda (L-curve), and one method to compute activation times. Previous computational studies have demonstrated that the relationship between signal noise and error in inverse reconstructions is dependent on the regularization and parameter selection methods used [12], [13]. We have demonstrated that signal processing (and therefore noise) does not dramatically change the inverse reconstruction, but rather has a major impact on the lambda value chosen (using an L-curve method) and therefore the accuracy of the inverse reconstruction. Different regularization parameter selection methods may be more or less sensitive to noise. Furthermore, the impact of different lambda values would change with different regularization methods, e.g. a higher lambda from the presence of baseline drift with first-order Tikhonov regularization would result in smoother reconstructions, while with BDR they would be more fractionated due to the smaller lambda values used. To test these hypotheses, we are currently running a variety of different methods on these data sets, which are also available for those who wish to test their own methods. The method to define activation times was based on a specific spatio-temporal approach combining the estimated time delays between neighboring electrograms and the standard maximum negative derivative approach [24]. Analysis using the standard derivative method to define activation produced the same trends as the spatio-temporal method, but the overall accuracy was worse due to the increase in activation time artefacts from high frequency noise. While we suspect the same trends would also be seen with other activation methods, these have not been assessed.

The signals on the heart and torso were recorded simultaneously for all data sets to avoid alignment issues and beat-to-beat variability. This means the noise present on the torso is also present in the epicardial signals and may bias the results. However, because the amplitude of the epicardial signals is much greater than on the torso, this bias does not impact the results of this study. To test this, we have compared reconstructions without filtering using only the 1^st^ and 18^th^ epicardial beats as the ground truth. The was nearly no difference in any metrics, e.g. the difference in absolute error was 0.03–0.07 mV higher than doing a beat-to-beat comparison, a change of less than 1.5%, and the correlation change was 0.2–1.4% reduced. There was no change for activation maps.

The study was conducted in an experimental setting, and it cannot fully represent the recording environment of a clinical setting. Here, only high-frequency noise from powerline interference and the baseline drift were considered. In a clinical environment, patient/electrode movement, breathing, and noise from muscles would also affect the signals. Despite this, we expect our results are directly transferable to the clinic. The channel white Gaussian noise present in the tank data sets are of a similar frequency to electromyogram noise. Likewise, the level of baseline drift present in the noisy tank data are of a similar level to that seen during breathing or patient movement, although morphologically different.

The sites of initial activation were determined automatically from activation maps. It is possible that an expert observer may have been able to identify sites of initial activation more precisely and that LE are over-estimated as a result. On the other hand, with our approach it was possible to analyze this large dataset efficiently and investigator bias was removed.

Finally, these methods were only evaluated in the presence of single-site pacing data. Given the low impact of signal processing on the inverse reconstruction, we expect the results of this study are applicable not only to stable “single” beat rhythms of clinical interest such as premature ventricular contractions or sinus rhythm, but also to the reconstruction of non-stable or multi-beat rhythms such as fibrillation or tachycardia, and for the reconstruction of repolarization. The question with these later cases is how to define the isoelectric point to ensure that the optimal lambda value is chosen.

## VIII. CONCLUSION

ECG signal processing has a fairly low impact on ECGI reconstruction accuracy. Removal of baseline drift improves electrogram reconstruction due to an improvement in the lambda selected for regularization, with a simple method of resetting the isoelectric point sufficient to see these improvements. High frequency noise removal does not impact electrogram reconstruction accuracy, but can improve post-reconstruction feature extraction. High frequency removal should be applied post-reconstruction with care to ensure over-filtering does not occur.
